# Delivery of Global Cancer Care: An International Study of Medical Oncology Workload

**DOI:** 10.1200/JGO.17.00126

**Published:** 2017-12-15

**Authors:** Adam Fundytus, Richard Sullivan, Verna Vanderpuye, Bostjan Seruga, Gilberto Lopes, Nazik Hammad, Manju Sengar, Wilma M. Hopman, Michael D. Brundage, Christopher M. Booth

**Affiliations:** **Adam Fundytus**, **Michael D. Brundage**, and **Christopher M. Booth**, Queen’s University Cancer Research Institute; **Nazik Hammad**, **Wilma M. Hopman**, **Michael D. Brundage**, and **Christopher M. Booth**, Queen’s University; **Wilma M. Hopman**, Kingston General Hospital Research Institute, Kingston, Canada; **Richard Sullivan**, King’s College London, King’s Health Partners Comprehensive Cancer Centre, London, United Kingdom; **Verna Vanderpuye**, Korle Bu Teaching Hospital, Accra, Ghana; **Bostjan Seruga**, Institute of Oncology, Ljubljana, Slovenia; **Gilberto Lopes**, University of Miami and Sylvester Comprehensive Cancer Center, Miami, FL; and **Manju Sengar**, Tata Memorial Centre, Mumbai, India.

## Abstract

**Background:**

To our knowledge, there is no literature that has described medical oncology (MO) workload in the global context. Here, we report results of an international study of global MO workload.

**Methods:**

An online survey was distributed through a snowball method via national oncology societies to chemotherapy-prescribing physicians in 65 countries. Countries were classified into low- or low-middle**–**income countries (LMICs), upper-middle**–**income countries (UMICs), and high-income countries (HICs) on the basis of World Bank criteria. Workload was measured as the annual number of new consultations provided to patients with cancer per oncologist.

**Results:**

A total of 1,115 physicians completed the survey: 13% (147 of 1,115) from LMICs, 17% (186 of 1,115) from UMICs, and 70% (782 of 1,115) from HICs. Eighty percent (897 of 1,115) of respondents were medical oncologists, 10% (109 of 1,115) were clinical oncologists, and 10% (109 of 1,115) were other. The median number of annual consults per oncologist was 175 (interquartile range, 75 to 275); 13% (140 of 1,103) saw ≥ 500 new patients in a year. Annual case volume in LMICs (median consults, 425; 40% of respondents seeing > 500 consults) was substantially higher than in UMICs (median consults, 175; 14% > 500) and HICs (median consults, 175; 7% > 500; *P* < .001). Among LMICs, UMICs, and HICs, median working days per week were 6, 5, and 5, respectively (*P* < .001). The highest annual case volumes per oncologist were in Pakistan (median consults, 950; 73% > 500 consults), India (median consults, 475; 43% > 500), and Turkey (median consults, 475; 27% > 500).

**Conclusion:**

There is substantial global variation in medical oncology case volumes and clinical workload; this is most striking among LMICs, where huge deficits exist. Additional work is needed, particularly detailed country-level mapping, to quantify activity-based global MO practice and workload to inform training needs and the design of new pathways and models of care.

## INTRODUCTION

Cancer is now the second leading cause of death worldwide. There is a disproportionately high burden in low- and low-middle–income countries (LMICs), where the mortality-to-incidence ratio is double that of high-income countries^1-3^ Although this is driven by a number of complex factors (including more advanced stage of disease at presentation), access to oncologists and the necessary infrastructure to deliver treatment are likely contributing factors. Cancer control efforts in LMICs are further challenged by the existing paradox in cancer funding; despite accounting for 62% of global cancer mortality, 5% of global cancer funding is directed to LMICs.^4^ It is therefore unlikely that mortality and incidence trends in LMICs will improve without a shift in global cancer policy.

Oncology workload metrics for LMICs are scarce. Limited data from high-income countries (HICs) have described clinical workload and proposed targets.^5-7^ However, this has not been done on a global scale and does not include LMICs. To develop an effective global cancer policy and bridge gaps in the delivery of cancer care, an understanding of global oncology workload is crucial. To address this gap in knowledge, we undertook a global study to describe the (1) clinical workload of medical oncologists, (2) available infrastructure and supports, and (3) identified barriers to patient care. Data from this study will inform cancer policy and human resource planning in emerging and established cancer systems.

## METHODS

### Study Population

The study population included any practicing physician who delivers chemotherapy; trainees were not eligible. The Web-based survey was distributed using a modified snowball methodology. As a means of identifying potential participants, the senior investigator (C.M.B.) contacted one oncologist in 54 countries and two regions (Caribbean and Africa) to invite study participation. Contact was preferentially directed to established national associations of medical oncologists. If this was not possible, C.M.B. approached one personal contact per country to invite participation and distribute the survey via an informal national network; this contact remained the sole source of survey distribution in the country. This study was approved by the Research Ethics Board of Queen’s University.

### Survey Design and Distribution

An online electronic survey questionnaire was developed via Fluid Surveys to capture the following information: participant demographics, clinical practice setting, clinical workload, and barriers to patient care. The survey was designed with multidisciplinary input of the study investigators who practice in diverse environments from LMICs, upper-middle–income countries (UMICs), and HICs. The survey was then piloted and subsequently revised based on feedback from 10 additional oncologists from diverse global backgrounds. The final survey included 51 questions and took 10 to 15 minutes to complete; the instrument is shown in the Data Supplement.

Distribution of this survey used two primary methods. The senior investigator (C.M.B.) contacted individuals and regional oncology associations to create a broad distribution network. Whether the regional contact was an association or an individual, they were provided with an electronic link to the survey to distribute to their regional membership/network. These links were unique to each nation, but not individualized. The distributing partners were asked to provide the team with the number of survey recipients to ascertain the national response rate for the survey. The survey was distributed in November 2016. A reminder e-mail was sent via all national/regional contacts in January 2017.

### Statistical Analysis

Countries were classified into LMICs, UMICs, and HICs on the basis of World Bank criteria.^8^ The primary objective was to describe oncologist workload across LMICs, UMICs, and HICs; oncologist workload was defined as the annual number of consultations for new patients with cancer seen per oncologist. Because of a relatively small number of responses from low-middle–income African nations, we combined these responses into a region called LMIC Africa. All data were initially collected in Fluid Surveys and subsequently exported to IBM Statistical Package for the Social Sciences (SPSS) for Windows version 24.0 (SPSS, Armonk, NY). Pearson χ^2^ tests were used to test for the difference in proportions, and the Kruskal-Wallis test was used to compare ordinal and continuous data by income stratification. Data consisted of categorical, ordinal, and continuous formats, occasionally collected as ranges (eg, < 50, 51 to 100, 101 to 150, etc). In the latter case, medians were generated using the midpoint of the categorical range (eg, a median value of 101 to 150 would be reported as 125). Data were analyzed using IBM SPSS.

## RESULTS

### Survey Distribution and Response

Fifty-four countries and two regional networks (Africa and Caribbean) were invited to participate in this study; 42 countries/regional networks (75%) agreed to participate. Among participating countries, the survey was distributed via national medical oncology organizations in 62% of cases (26 of 42) and via an informal network of contacts in 38% of cases (16 of 42). Overall, 1,115 respondents from 65 different countries participated in this study. Survey response rates were available for 40% (17 of 42) of all countries/regional networks and ranged from 3% in Singapore and Portugal to 76% in Slovenia (Data Supplement). Among study participants, 70% (782 of 1,115), 17% (186 of 1,115), and 13% (147 of 1,115) were from HICs, UMICs, and LMICs, respectively. The mean response rate across all countries was 12% (461 of 3,967); it was 12% (30 of 255), 13% (30 of 235), and 12% (401 of 3,477) for LMIC, UMIC, and HIC countries, respectively (*P* = .85).

### Characteristics of Study Participants

The median age of respondents was 44 years; 58% (647 of 1,110) were male (Table 1). The proportion of female respondents was higher in HICs (44%; 341 of 777) and UMICs (47%; 87 of 186) compared with LMICs (24%; 35 of 147; *P* < .001). Eighty-one percent (898 of 1,115) of all respondents were medical oncologists; the median number of years in practice was 10, with a median of 6 years of postgraduate training. Participants from LMICs were more likely to be clinical oncologists (ie, delivering chemotherapy and radiation; 20%; 29 of 147) than were those from UMICs (9%; 16 of 186) and HICs (9%; 67 of 782; *P* < .001). Participants in LMICs were less likely to have completed training in their current country of practice (82%; 120 of 147) compared with UMICs (91%; 170 of 186) and HICs (90%; 707 of 782; *P* = .004).

**Table 1:**
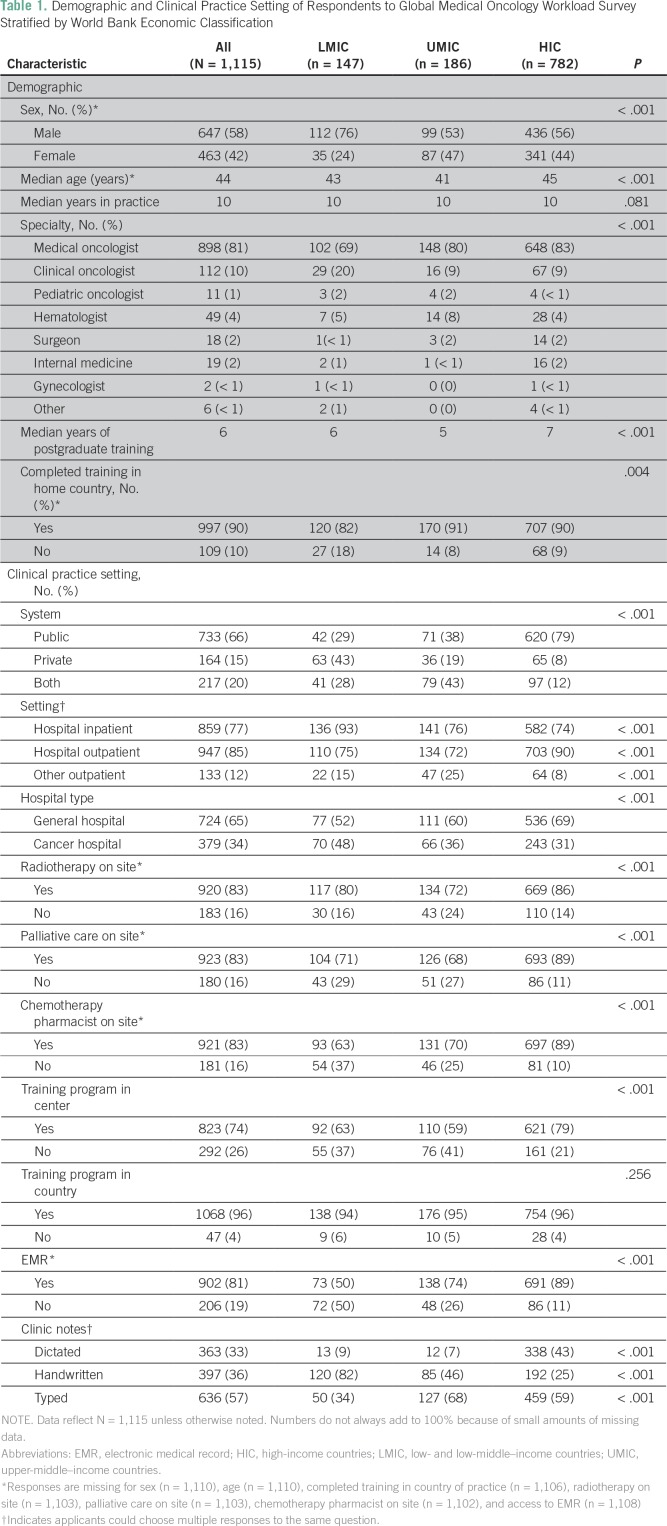
Demographic and Clinical Practice Setting of Respondents to Global Medical Oncology Workload Survey Stratified by World Bank Economic Classification

### Clinical Practice Setting

The proportion of respondents working exclusively in the public setting varied substantially: 29% (42 of 146) in LMICs, 38% (71 of 186) in UMICs, and 79% (620 of 782) in HICs (*P* < .001). Physicians in LMICs were more likely to work in a designated cancer hospital (48%; 70 of 147) compared with UMICs (36%; 66 of 186) and HICs (31%; 243 of 782; *P* < .001). Respondents from LMICs (39%; 58 of 147) were more likely to work within a smaller group (more than five) of chemotherapy providers compared with UMICs (26%; 48 of 186) and HICs (10%; 76 of 782; *P* < .001). On site radiation, palliative care, and chemotherapy pharmacists were less likely to be available at LMIC centers (80% [117 of 147], 71% [104 of 147], 63% [93 of 147] availability, respectively) compared with HICs (86% [669 of 782], 89% [693 of 782], 89% [697 of 782] availability, respectively; all *P* < .001). Electronic medical records were available less commonly in LMICs (50% [73 of 147] *v* 89% [691 of 782]; *P* < .001), and corresponding rates of handwritten clinic notes were much higher in LMICs compared with UMICs and HICs (82% [120 of 147] for LMICs *v* 46% [85 of 186] for UMICs and 25% [192 of 782] for HICs; *P* < .001).

### Delivery of Clinical Care

LMIC respondents worked a median of 6 days per week, whereas both UMIC and HIC respondents reported working a median of 5 days per week (*P* < .001); 71% (104 of 147) of LMIC physicians worked 6 to 7 days per week compared with 21% (166 of 782) of HIC physicians. Median hours worked per week were 41 to 50 across all groups. LMIC and UMIC respondents reported a median of 4 and 3 weeks of paid vacation per year, respectively, compared with 5 weeks for HIC respondents (*P* < .001); 20% (29 of 147) of LMIC and 3% (23 of 782) of HIC physicians had no paid vacation. The median number of weeks of paid conference leave and the proportion of physicians with no paid conference leave for LMICs, UMICs, and HICs was 2 weeks (29%; 43 of 147), 1.5 weeks (20%; 37 of 186), and 2 weeks (10%; 77 of 782), respectively (*P* < .001). Although there was no substantial difference in the proportion of respondents who had on-call duties (68% [100 of 147], 63% [117 of 186], 72% [565 of 782] for LMIC, UMIC, and HIC, respectively); oncologists who took call in LMICs were more likely than UMIC or HIC physicians to be on call every night except when on vacation (60% [59 of 99] *v* 41% [48 of 116] and 17% [96 of 560]; *P* < .001). The mean percentage of time that study respondents spent on clinical, research, teaching, and administrative duties were consistent across the three groups (Table 2).

**Table 2:**
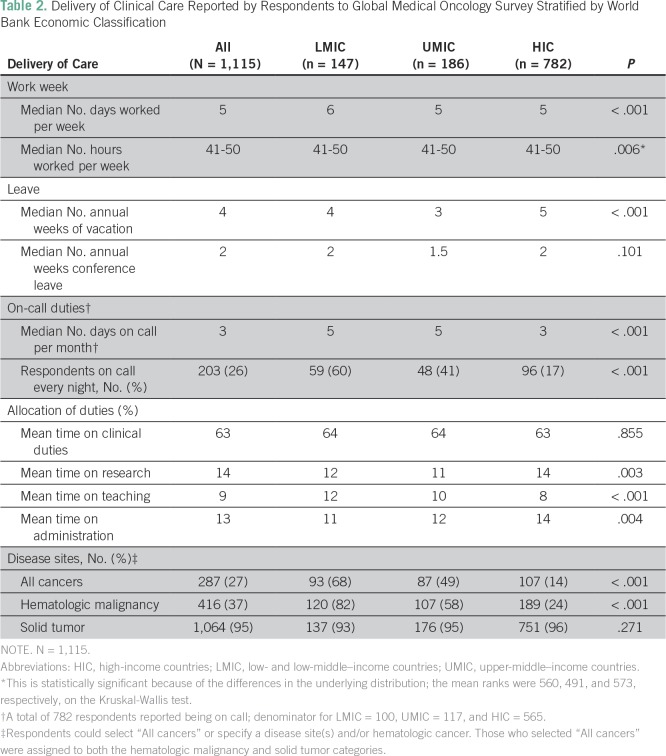
Delivery of Clinical Care Reported by Respondents to Global Medical Oncology Survey Stratified by World Bank Economic Classification

### Clinical Volumes

**Fig 1:**
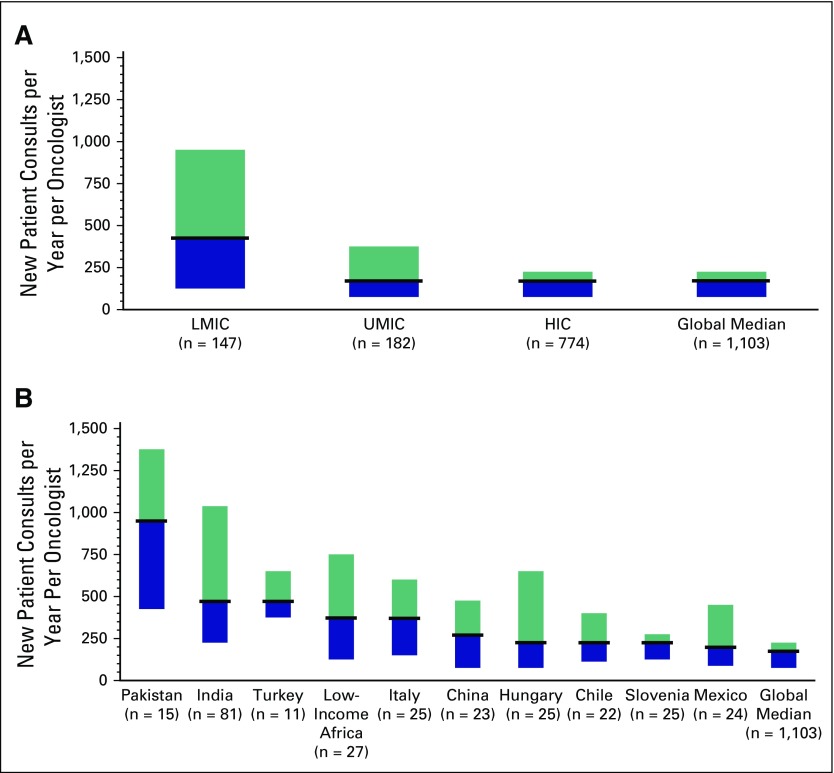
Median annual new patient consultations (with 25th to 75th percentile) reported by 1,115 oncologists globally. Results are shown (A) by World Bank economic status and (B) for the highest volume countries globally. LMIC, low-middle–income country.

The median number of new consults per year among all respondents was 175; 13% (140 of 1,103) saw > 500 and 6% (69 of 1,103) saw > 1,000 new consults per year. Respondents from LMICs reported seeing significantly more consults (median, 425/y) than UMIC and HIC respondents (median, 175/y; *P* < .001). The proportion of oncologists in LMICs seeing > 500 (39%; 58 of 147) and > 1,000 (22%; 33 of 147) new consults was substantially higher than in UMICs (14%, 25 of 182; and 6%,11 of 182, respectively) and HICs (7%, 57 of 774; and 3%, 25 of 774, respectively; *P* < .001). Distribution of clinical workload across economic groups and among the top 10 countries is shown in Figure 1. The 10 highest-volume countries were Pakistan (975; 73% > 500 new consults), India (475, 43% > 500), Turkey (475; 27% > 500 new consults), LMIC Africa (375; 37% > 500 new consults), Italy (325; 32% > 500), China (275; 22% > 500), Hungary (225, 29% > 500), Slovenia (225; 12% > 500), Chile (225; 9% > 500), and Mexico (200; 21% > 500)

The number of patients seen in a full day of clinic varied across economic groups (LMIC, 25; UMIC, 25; HIC, 15; *P* < .001); 20% (30 of 147) of LMIC oncologists saw > 50 patients per day compared with 2% (12 of 774) in HICs (*P* < .001). Oncologists in LMICs were considerably more likely to treat all tumor types compared with those in UMICs and HICs (68% *v* 49% *v* 14%; *P* < .001). LMIC respondents reported less time per patient interaction (25 minutes per new consult) compared with UMIC and HIC respondents (35 minutes; *P* < .001). Wait time for new consults to be seen (measured from time of referral) was significantly shorter in LMICs (median wait, 0 days) compared with UMICs and HICs (4 to 7 days for each; *P* < .001); 56% (83 of 147) of LMIC oncologists reported seeing patients on the same day of referral/presentation. Participation in multidisciplinary case conferences varied across economic groups; 54% (80 of 147) of LMIC and 50% (93 of 186) of UMIC oncologists attended at least one multidisciplinary case conference per week compared with 80% (627 of 782) of HIC oncologists (*P* < .001; Table 3).

**Table 3:**
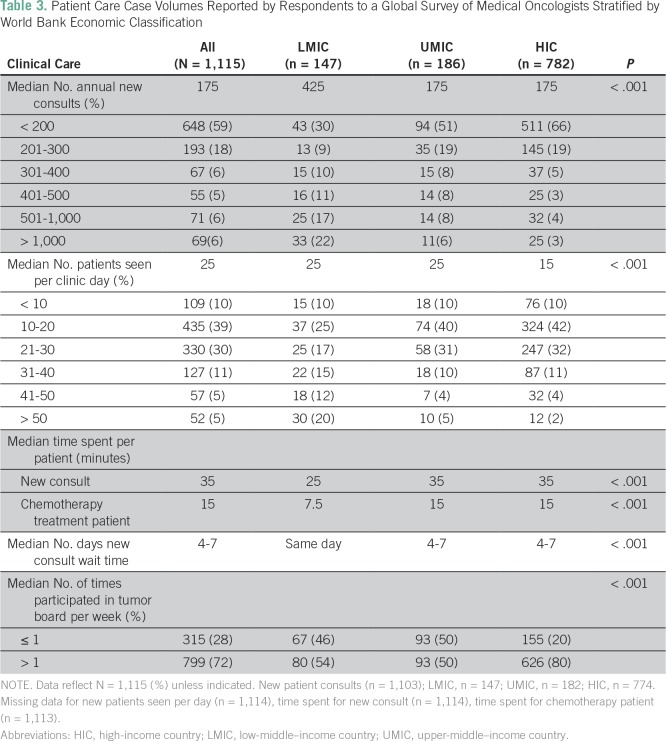
Patient Care Case Volumes Reported by Respondents to a Global Survey of Medical Oncologists Stratified by World Bank Economic Classification

### Satisfaction, Barriers, and Challenges

Self-reported job satisfaction (on a Likert scale; 1 = not satisfied, 10 = highly satisfied) did not vary across economic groups (median score, 8 in all groups). Despite lower clinical volumes, physicians in HICs (68%; 529 of 780) and UMICs (75%; 139 of 186) were more likely than oncologists in LMICs (52%; 76 of 147) to report high patient volumes as adversely affecting job satisfaction (*P* < .001). The most commonly reported barriers to clinical care in LMICs were patients not being able to pay for treatment and limited availability of new cancer therapies. The most common barriers reported in HICs were high clinical volumes and insufficient time to keep up with published literature (Table 4).

**Table 4:**
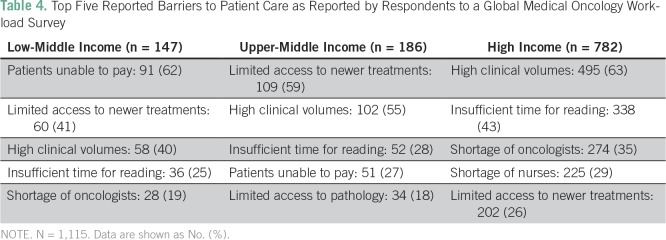
Top Five Reported Barriers to Patient Care as Reported by Respondents to a Global Medical Oncology Workload Survey

## DISCUSSION

This study offers insights into the clinical practice setting and workload of medical oncologists working in different contexts and resource settings. Several important findings emerge. First, there is a substantial difference in clinical workload across economic settings; oncologists in LMICs see significantly more patients, work more days, are more often on call, and have less vacation time than their global counterparts. Second, oncologists in LMICs are less likely to work in the public system and have less access to parallel cancer services, such as radiotherapy, palliative care, and multidisciplinary team meetings, than oncologists in UMICs and HICs. Third, the higher clinical volumes in LMICs are associated with less time spent with patients. Finally, we observed a disconnect between clinical volume and the reported barriers to patient care. Despite substantially lower patient volumes, oncologists in HICs and UMICs identify high clinical workload as a top barrier to patient care; the top barriers identified by oncologists in LMICs relate to patients being unable to pay for care and limited access to cancer therapies.

Our study results should be considered in light of existing literature on this topic. Our data confirm anecdotal reports that specialist case volumes in LMICs are substantially higher than in UMICs and HICs.^9^ Two recent studies have reported oncology workloads in HICs. In 2012, Blinman et al^7^ described a survey of 96 Australian medical oncologists reporting a mean 270 new patient consults per year. A 2013 survey of 33 New Zealand medical oncologists reported 220 new patient consults per year.^6^ These data are slightly higher than our own median value of 175 consults per year in HICs (and 175 in Australian respondents, specifically).

The Systemic Therapies Task Force established by Cancer Care Ontario in 2000 determined that 160 to 175 was the optimal annual target for medical oncology new consults.^5^ This number was derived by calculating the annual amount of hours per oncologist per year available for direct patient care and then dividing this number by a tumor-specific patient care time to calculate the number of annual new patient consults. The tumor-specific patient care time comprised the total number of hours that an oncologist should expect to dedicate to an average new patient for each tumor type over a 5-year period.^5^ Although the LMIC data in our study (425 consults per year) were substantially above this target, self-reported workload of UMIC and HIC respondents fell within this recommended range.

Despite seeing much higher volumes than their UMIC and HIC counterparts, a smaller proportion of our LMIC respondents listed high clinical volumes as a barrier to care. This highlights the fact that although LMIC nations likely have a shortage of oncologists, the delivery of cancer care in low-resource settings presents multifactorial challenges, with fundamental economic barriers being a more pressing issue than practitioner shortage. Accordingly, our data suggest that a standardized model of cancer care cannot be applied equally to LMIC, UMIC, and HIC countries and that an individualized approach is required.

Workload studies do exist in the field of radiation oncology. A recent European working group recommended a maximum number of consults per year of 250 for radiation oncologists.^10^ Previous radiation oncology workload studies from Japan (n = 194 to 291 annual new consults), Australia (n = 250), and Thailand (n = 296) suggest slightly higher new consult loads compared with medical oncologists.^11-13^ However, direct comparisons between medical oncology and radiation oncology new consult targets are of limited utility because the physician-level and system-level workload are different in each setting.

Existing literature on oncologist burnout provides a basis for comparison with some of our data. Shanafelt et al^14^ examined burnout and job satisfaction in a 2014 survey of 11,117 American oncologists. Compared with this study, our participants were younger (45 *v* 52 years), more recently in practice (10 *v* 22 years), and worked a comparable number of hours per week (41 to 50 *v* 46).^14^ HIC respondents in our study reported less time with new patients (35 minutes *v* 52 minutes). The Cancer Care Ontario analysis reported a comparable number of hours worked (48 hours per week).^5^ Glasberg et al^15^ completed a study of burnout among 102 Brazilian oncologists in 2007; they reported comparable working hours (< 50) to our UMIC respondents (41 to 50). The consistency between workload metrics in the aforementioned studies from the United States, Canada, Australia, and Brazil and workload reported by our HIC and UMIC respondents offers face validity to the results of our global study.

Our study results should be considered in light of methodologic limitations. As with any survey, respondents may not be representative of all providers in each system. Our results are further limited by the fact that 16 of 42 countries did not have a national association and relied on informal survey distribution by one contact oncologist. We also were unable to identify the denominator (ie, response rate) for many countries (Data Supplement). It is, however, reassuring that the response rate was comparable across LMICs, UMICs, and HICs. Workload data are self-reported and therefore may or may not accurately reflect true clinical volumes. Our study has a limited number of respondents from very low-income countries. We are also missing data from the United States and Russia; two of the world’s largest countries chose not to participate in this study. The LMIC group had the lowest number of respondents in our survey, indicating the difficulty of reaching this population of oncologists. Building on our results will require country-level analysis using more sophisticated sampling instruments to guide policy recommendations. Our results also provide comparative data that may be useful for individual health systems. Finally, delivery of systemic therapy is only one element of cancer care, and meaningful improvements in cancer care will require parallel initiatives in other allied clinical disciplines, such as radiation/surgical oncology, palliative care, pathology, radiology, nursing, and pharmacy.

Health care human resource (HHR) planning has been belatedly recognized as critical to achieving universal health coverage and the health targets of Sustainable Development Goals of the WHO. Most empirical work has been focused at the macro-level of HHR planning. There is uniform agreement that a demand-based shortage of 15 million or more health care workers will be the reality by 2030, with shortages being most acutely felt in middle-income countries, as well as East Asia and the Pacific.^16^ This crisis of human capital in health is one of availability (supply of qualified personnel), distribution (recruitment, retention where needed most), and performance (productivity and quality of care provided). There is, however, a dearth of cancer-specific HHR research. What has been done in surgery^17^ and radiotherapy^18^ has primarily focused on using worker-to-population ratios that ignore need, demands, and institutional frameworks. More focused HHR studies in cancer at the country level have also suffered from overmodeling and a lack of real-world data. However, even country-level data concur.^19^ The deficits among need, demand, and provision are wide and widening. This presents a fundamental challenge to the ability of global cancer to deliver its universal health coverage and Sustainable Development Goal commitments. The real-world data presented in our current work provide one aspect of a multimethodologic approach needed to study cancer HHR to inform policy. To drive changes in cancer HHR policy, a variety of supply-and-demand methods (needs-based, utilization or demands-based, workforce-to-population ratios, and target setting) will be required. Cancer care has one of the most complex HHR patterns in health care, and national-level studies are crucial to accurately inform long-term planning.^20^

In summary, we report substantial global variation in medical oncology case volumes and clinical workload; this is most striking among LMICs, where huge deficits exist. Additional work is needed, particularly detailed country-level mapping, to quantify activity-based global medical oncology practice and workload to inform training needs and the design of new pathways and models of care.
